# Distribution of the polycystine radiolarian species in the Quaternary sediment cores of the subarctic North Atlantic and Sea of Okhotsk

**DOI:** 10.1016/j.dib.2018.01.041

**Published:** 2018-02-02

**Authors:** Alexander Matul

**Affiliations:** Shirshov Institute of Oceanology, Russian Academy of Sciences, Nahimovskiy prospekt 36, 117997 Moscow, Russia

**Keywords:** Quaternary sediment cores, Marine micropaleontology, Polycystine radiolarians, North Atlantic, Sea of Okhotsk

## Abstract

Data files present information on the quantitative distribution of the polycystine radiolarian species in the Quaternary marine sediments from the subarctic areas. Dataset on the open North Atlantic north of 43 °N contains percentages of 35 abundant (> 1%) species in 145 samples of five sediment cores. Dataset on the Sea of Okhotsk contains percentages of 31 abundant (> 1%) species in 794 samples of seven sediment cores. Dataset on the bottom surface sediments of the North Atlantic contains percentages of 35 abundant (> 1%) species in 78 grab and core-top samples. For the microscopic radiolarian analysis, the sediment fraction of > 40/50 μm was used.

**Specifications Table**

**Table:** 

Subject area	*Earth Sciences*
More specific subject area	*Marine micropaleontology*
Type of data	*Tables*
How data was acquired	*Microscope*
Data format	*Raw (percentages of species content)*
Experimental factors	*Standard laboratory treatment of the marine sediment samples using the hydrogen peroxide, sodium pyrophosphate, and Canada balsam as mounting media for slides preparation*
Experimental features	*As a rule, at least 300–350 radiolarian tests were counted per one slide*
Data source location	*P.P. Shirshov Institute of Oceanology, Moscow, Russia*
Data accessibility	*Data is with this article*

**Value of the data**

• data on the distribution of the microfossils in the oceanic bottom sediments provide an information on the development of the environments and climate as the marine microorganisms are valuable environmental indicators
• we publish the detailed micropaleontological datasets on the subpolar areas of the Northern hemisphere where the long- to short-term climatic changes exhibit a high magnitude
• our micropaleontological data are obtained according the standard analytical technique so that they can be used without significant modification by other specialists
• main research output from use of our datasets is a reconstruction of paleoenvironments (based on factor, cluster, correspondence analysis), such as water temperature, to define trends and possible cycling of the paleoclimatic changes

## 1 Data

**Fig. 1:**
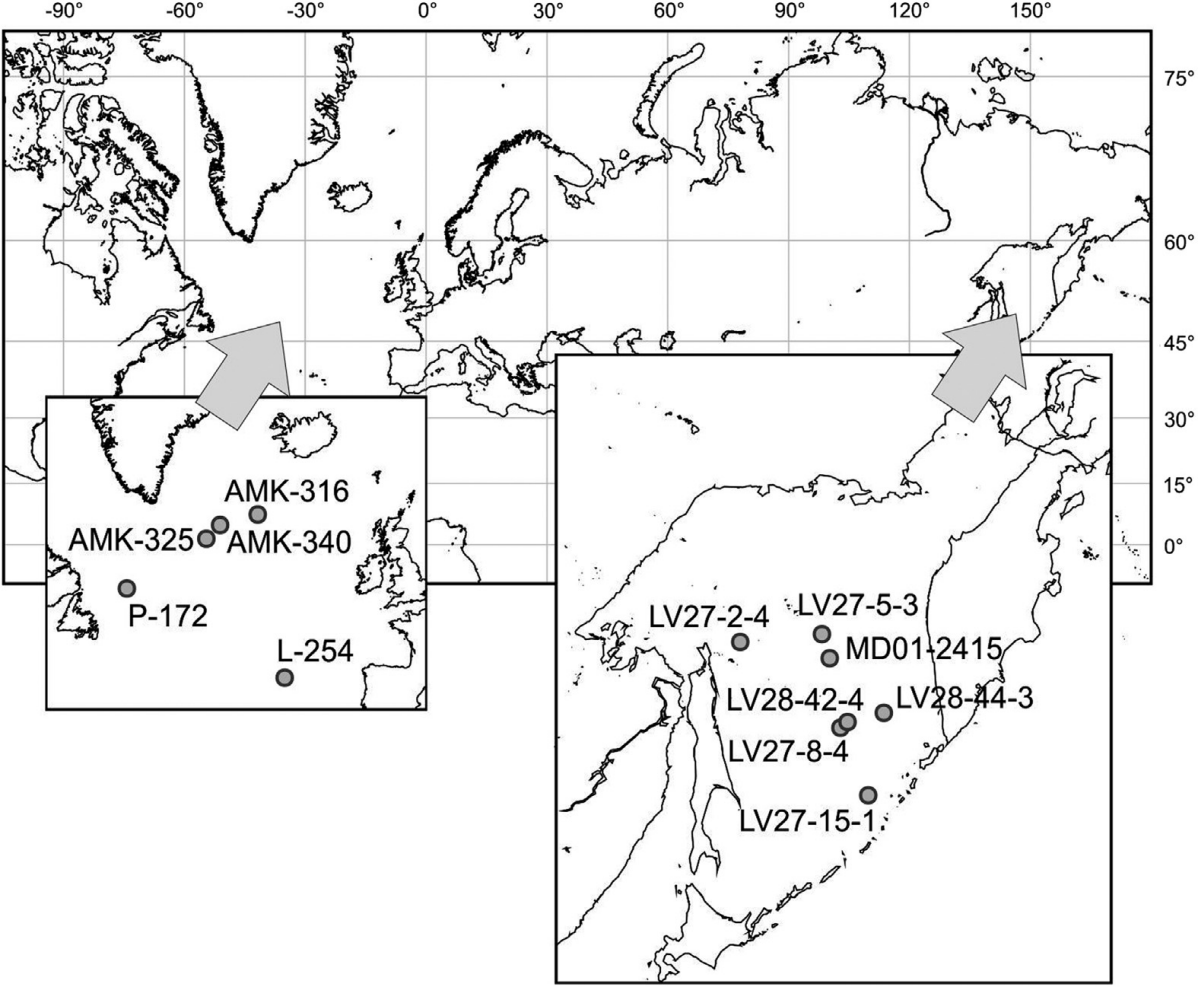
Location of the sediment cores which are provided with data files.

Data files contain the quantitative information on the relative content (%) of the abundant (> 1% of concentration) species of the polycystine radiolarians in the Quaternary sediments of the subarctic North Atlantic and Sea of Okhotsk. Polycystine radiolarians as protists with silica skeleton are one of major groups of the marine microplankton. They can be accumulated in great numbers on the oceanic floor, and then extracted from sediments for the biostratigraphic and paleoceanographic studies. The Quaternary sediments contain diverse lithophysical, geochemical, and micropaleontological information on the glacial to interglacial paleoclimatic changes. We provide detailed micropaleontological data mostly on the last glacial cycle (last interglacial to last glacial to Holocene as recent interglacial) in 5 sediment cores from the northern North Atlantic (35 radiolarian species in 145 samples) and 7 sediment cores from the Sea of Okhotsk (31 radiolarian species 794 samples) (Fig. 1; Table 1). Data were used previously in publications on the Quaternary paleoceanography of the North Atlantic [[1], [2], [3]] and North Pacific [[4], [5], [6], [7]]. Also data on the radiolarian distribution in the bottom surface sediments of the open North Atlantic are presented.

**Table 1:** Location of sediment cores.

**Sediment core**	**Latitude, N**	**Longitude, E, “-“ is W**	**Water depth, m**	**Area**
L-254	43,7883	−22,7667	3950	North Atlantic
AMK-316	58,735	−27,2883	2155	North Atlantic
AMK-325	58,4367	−31,7033	1820	North Atlantic
AMK-340	58,51	−31,52	1699	North Atlantic
P-172	53,3167	−47,15	3420	North Atlantic
LV27-2-4	54,5025	144,7523	1301	Sea of Okhotsk
LV27-5-3	54,7682	149,4985	476	Sea of Okhotsk
LV27-8-4	51,5053	150,5715	1160	Sea of Okhotsk
LV27-15-1	49,0002	152,1938	1991	Sea of Okhotsk
LV28-42-4	51,7148	150,9854	1041	Sea of Okhotsk
LV28-44-3	52,0419	153,0992	684	Sea of Okhotsk
MD01-2415	53,9515	149,9587	822	Sea of Okhotsk

## 2 Experimental design, materials and methods

The laboratory treatment of sediment samples (1–2 g of dry bulk sediment) and preparation of the radiolarian slides for the microscopic analysis was made the standard technique [8]: cleaning of the radiolarian skeletons while boiling of the sediment sample in solutions of hydrogen peroxide and sodium pyrophosphate, sieving of residue through the mesh of 40 μm (for the Sea of Okhotsk sediment samples), and of 50 μm (for the North Atlantic sediment samples), settling of radiolarian tests on the slide in the Petri dish, mounting of radiolarian tests on slides in the Canada balsam or Mountex. At least 300–350 radiolarian tests were counted per one slide to calculate the total radiolarian abundances and relative concentrations of radiolarian species.
